# Recurrent Bouts of Fever and Transient Hydrosalpinx Manifested in a Female Carrying MEFV G304R Gene Variant: A Case Report

**DOI:** 10.7759/cureus.55188

**Published:** 2024-02-28

**Authors:** Takashi Aikawa, Shohei Yoshida, Kouki Saruwatari, Yuko Hasegawa, Issei Kagami

**Affiliations:** 1 Internal Medicine, Kiryu Kosei General Hospital, Kiryu, JPN; 2 Obstetrics and Gynecology, Kiryu Kosei General Hospital, Kiryu, JPN

**Keywords:** colchicine, mefv exon2 g304r heterozygous, transvaginal sonography, hydrosalpinx, familial mediterranean fever

## Abstract

Familial Mediterranean fever (FMF) is an inherited autoinflammatory disease characterized by recurrent bouts of fever and serositis. Mediterranean Fever (*MEFV*) gene mutations may cause not just FMF but various serositis including arthritis, enterocolitis, aseptic meningitis, pulmonary disease, and pericarditis. In this report, we present a 44-year-old female carrying *MEFV* gene variant. She was admitted to our hospital with a high fever, right back pain during inspiration, and lower-left abdominal pain. Laboratory findings showed high inflammatory response. Computed tomography (CT) indicated pleurisy of the right lobe and inflammation of the left uterine appendage. Transvaginal sonography and magnetic resonance imaging (MRI) indicated hydrosalpinx of the left oviduct. The symptoms of recurrent fever and transient serositis suggested FMF, and abdominal pain was resolved after taking colchicine. Later, it turned out that she had *MEFV* gene mutation (exon2 G304R heterozygous). Although she did not meet the criteria of FMF, this is the first reported *MEFV* variant carrier with transient hydrosalpinx. Attacks in female patients with FMF are triggered by menstruation. Moreover, FMF and associated amyloidosis may cause both male and female infertility. Although male patients with FMF may present with acute scrotum, diagnostic criteria of FMF do not include inflammation of uterine appendages. Internal medicine physicians need to cooperate with gynecologists to diagnose female patients carrying *MEFV* gene variants.

## Introduction

Familial Mediterranean fever (FMF) is an inherited autoinflammatory disease characterized by recurrent bouts of fever and serositis [1]. FMF is caused by MEFV gene mutations on chromosome 16. The *MEFV *gene encodes for pyrin protein, which is a part of the pyrin inflammasome. Mutated forms of pyrin result in the impaired assembly of the inflammasome, leading to excessive production of proinflammatory cytokines [2,3].

As its name suggests, FMF is seen primarily among individuals of Mediterranean ancestry, particularly non-Ashkenazi Jews, Armenians, Arabs, and Turks. However, the prevalence of *MEFV* gene variants is not very low in East Asians [2,3]. Patients carrying *MEFV *gene variants may manifest not just FMF but various serositis including enterocolitis, pericarditis, pleurisy, and aseptic meningitis.

Attacks of patients with FMF are triggered by specific factors (e.g., psychological stress, tiredness, or menstruation), and some female patients with FMF manifest abdominal tenderness triggered by menstruation [4]. Moreover, FMF and associated amyloidosis may cause both male and female infertility. However, the details of triggers and infertility remain unknown.

We report a case in which a female patient with *MEFV* gene variant presented with transient hydrosalpinx caused by adnexal serositis.

## Case presentation

A 44-year-old Japanese female presented with fever (≥38℃) and lower abdominal tenderness that started two days before. She started to feel nausea and her vaginal discharge turned bloody on the day of presentation. Laboratory tests done in a nearby medical clinic indicated high inflammation response and renal insufficiency. She was referred to our emergency room for further investigation. Her medical history included hypertension, hyperlipidemia, and hyperuricemia with infrequent gout flares. Her renal function had deteriorated from what it was three years before the presentation. Her former values of creatinine were 1.12mg/dL (three months before), 0.94mg/dL (one year before), 0.71mg/dL (two years before), and 0.57mg/dL (three years before). She had no significant family history of periodic fever or abdominal pain. Medication taken by the patient included amlodipine, azosemide, pemafibrate, febuxostat, and iIcosapent ethyl. The obstetrics and gynecology questionnaire revealed that she had dysmenorrhea with menorrhagia, her previous menstrual period started 19 days prior to presentation, and her last sexual intercourse was 10 days earlier.

On presentation, the patient’s vital signs were as follows: body (axillary) temperature of 38.3°C, pulse rate of 120 beats per minute, blood pressure of 128/78 mmHg, and oxygen saturation of 90% on ambient air. She had lower-left abdominal tenderness and rebound tenderness. Crackles or wheezes were not heard on auscultation. The patient had spontaneous pain in her right back accompanying inspiration.

**Figure 1:**
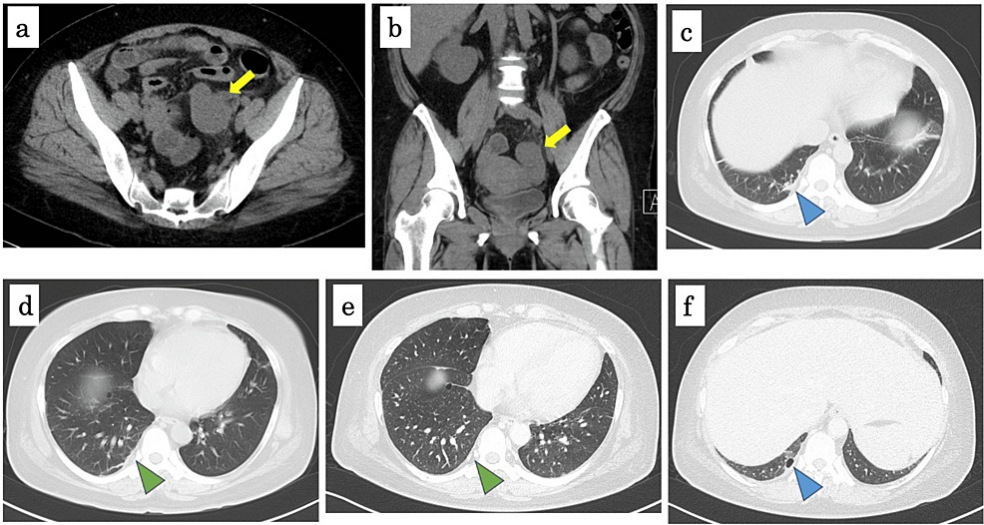
CT on admission (a-d) and on day 5 after admission (e-f) (a, b) Abdominal CT on admission demonstrated that the left side of uterine appendages was enlarged (yellow arrows); (c, d) Chest CT on admission demonstrated that small consolidation was located beside the pleura of the lower lobe of the right lung (blue arrow) and the pleura was thickened mildly (green arrow); (e, f) Chest CT on day 5 after admission demonstrated that the pleura was not thickened (green arrow) and the consolidation turned to bulla (blue arrow).

**Figure 2:**
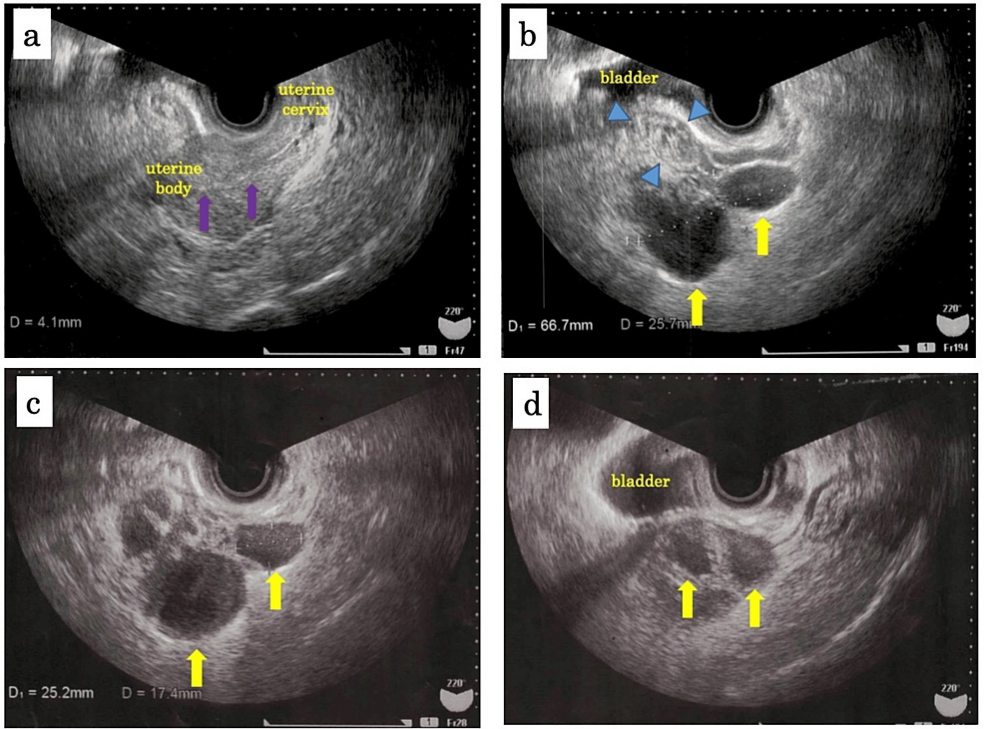
Transvaginal sonography on admission (a-b), on day 7 (c), and on day 18 (d) after admission (a, b) Transvaginal sonography on admission showed the endometrium (4.1 mm) was not thickened (purple arrows), the left ovary was not enlarged (blue arrows), and the left oviduct was enlarged and distorted resembling consecutive cysts (yellow allows); (c) Transvaginal sonography on day 7 after admission showed no significant change in the left oviduct (yellow allows); (d) Transvaginal sonography on day 18 after admission showed that the left oviduct had been smaller than at admission (yellow allows).

The laboratory evaluation showed an inflammatory response, iron-deficiency anemia, and acute renal failure (Table 1). Urine examination indicated pre-renal acute kidney injury, rather than the urinary tract infection or albuminuria (Table 2). Computed tomography (CT) demonstrated that the left side of the uterine appendages was enlarged (Figure 1a, 1b). CT also demonstrated that a small consolidation was located beside the pleura of the lower lobe of the right lung, and the pleura was thickened mildly (Figure 1c, 1d). The gynecologist found abnormal uterine bleeding on internal examination. Transvaginal sonography showed the endometrium (4.1 mm) was not thickened, and that the left oviduct was more enlarged and distorted than the right one (Figure 2a, 2b). ER doctors suspected sepsis from pelvic inflammatory disease (PID). Subsequently, the patient was admitted and received antibiotic therapy.

**Table 1:** Laboratory findings after admission eGFR: estimated glomerular filtration rate; UIBC: unsaturated iron binding capacity; PT-INR: prothrombin time-international ratio; FDP-D-dimer: fibrinogen degradation products fragment D dimer

Tests	Day 1-2	Day 138
Complete blood cell		
White blood cell (/μL)	10400	3700
Neutrophil (/μL)	7300	1800
Lymphcyte (/μL)	700	1480
Eoinophil (/μL)	31	140
Hemoglobin (g/dL)	9.3	9.4
Hematocrit (%)	27.7	29.7
Mean corpuscular volume (fL)	91.9	89.5
Platelet count (x10^4/μL)	32.5	30.9
Biochemistry		
Total protein (g/dL)	7.0	7.1
Albumin (g/dL)	2.9	4.0
Lactate dehydogenase (IU/L)	124	144
Creatine phosphokinase (U/L)	23	37
Total bilibubin (g/dL)	0.7	0.3
Aspartate aminotransferase (IU/L)	11	27
Alanine aminotransferase (IU/L)	13	20
Alkaline phosphatase (IU/L)	69	93
γ-glutamyl transpeptidase (IU/L)	141	120
Uric acid (mg/dL)	10.9	11.4
Blood urea nitrogen (mg/dL)	46	19
Creatinine (mg/dL)	2.88	1.18
eGFR (mL/min/1.73^2)	15.2	40.1
Sodium (mEq/L)	137	145
Chloride (mEq/L)	99	109
Potassium (mEq/L)	3.0	4.2
C-reactive protein (mg/dL)	42.2	0.04
Serum amyloid A (μg/mL)	2570	< 2.0
Iron (μg/dL)	6	65
UIBC (μg/dL)	275	307
Ferritin (ng/dL)	167.5	16.7
Coagulation test		
PT-INR	1.4	1.03
FDP-D-dimer (μg/mL)	1.32	0.24
Genetic testing		
Human leukocyte antigen	A-24, A-33, B-61, B-44	
MEFV	exon2 c.910G>A, p.Gly304Arg, heterozygous	

**Table 2:** Urine examination after admission

Tests	Day 1	Day 138
Urine dipstick test		
pH	5.5	5.5
Specific gravity	1.01	1.01
Protein	1+	2+
Glucose	-	-
Ketones	-	-
Bilirubin	-	-
Blood	3+	3+
White blood cells	-	-
Biochemistry		
Miroalbuminuria (mg/L)	129.9	
Protein (mg/dL)		126
Creatinine (mg/dL)	60.82	48.7
Sodium (mEq/L)	36	
Potassium (mEq/L)	8.0	
Urine urea nitogen (mg/dL)	307	
β2-microglobulin (mg/L)	10.59	1.77
N-acetyl-beta-d-glucosaminidase activity (U/L)		3.6
Microscopic examination		
Organisms	-	-
Hyaline cast	+	-

**Figure 3:**
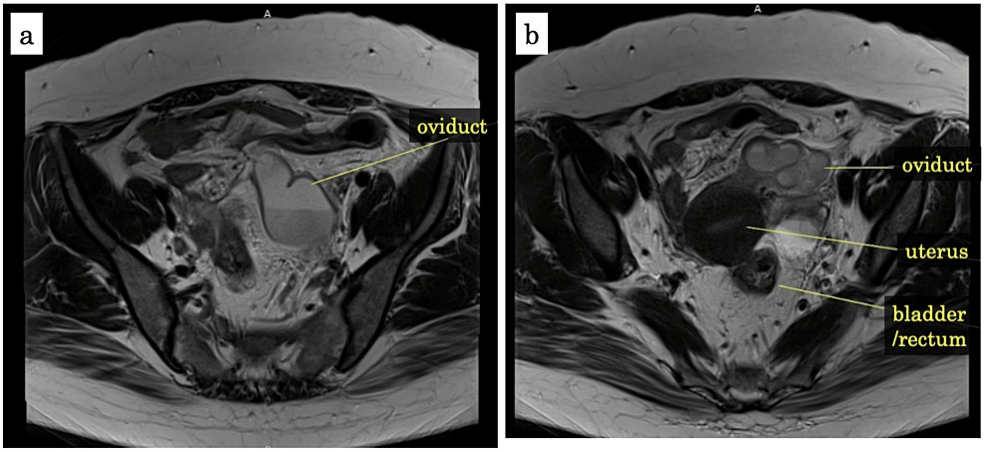
MRI on day 4 after admission MRI demonstrated that the fluid level was inside the left oviduct, and the left oviduct was enlarged like consecutive cysts.

On day 4 after admission, the gynecologist found mild tenderness around the left uterine appendages; however, the tenderness had become less than it was on admission. Transvaginal sonography showed the left oviduct was still enlarged (Figure 2c). Magnetic resonance imaging (MRI) demonstrated hydrosalpinx in the left oviduct, and the left oviduct was enlarged resembling consecutive cysts (Figure 3). These findings suggested that she did not have endometriosis, and the left hydrosalpinx had been probably caused by the inflammation of uterine appendages. The bacteria cultures from blood, urine, and vaginal secretion were negative. Vaginal swab tests for chlamydia and gonorrhoeae were negative. She started food intake on day 2. Her body temperature got back to normal on day 3. Chest CT on day 5 demonstrated that the pleura was no longer thickened and the consolidation turned to bulla (Figure 1e, 1f). Further history-taking revealed that she sometimes had a high fever prior to her menstrual period. In addition, she had gout flares affecting her ankle and took colchicine several times. Due to periodic fever along with menstruation and arthralgia of the ankle, she was suspected of FMF and was started on colchicine (1.0 mg/day) on day 5. She was discharged on day 7 after admission.

After discharge, she visited the gynecologist and the physician for follow-up. The gynecologist confirmed recession of abdominal tenderness and alleviation of the left hydrosalpinx with transvaginal sonography on days 18 and 67 after discharge (Figure 2d). Twenty-two days after the discharge, she suffered from lower abdominal tenderness for two days. However, it was resolved after the menstruation began. Again, 41 days after her discharge, recurrent bouts of fever (≥39℃) occurred without abdominal tenderness for two days.

Genetic analysis revealed that she had the G304R heterozygous mutation in exon 2 of* the MEFV* gene. She did not match the Livneh Criteria [5], because the exon 2 variant of *MEFV* is not necessarily the pathogenesis of FMF, she had no family history of FMF, and the symptoms were not necessarily periodic. However she manifested the symptoms of serositis in a row, and she had a favorable response to colchicine. Therefore, we diagnosed her with *MEFV* gene-related disease.

The patient did not suffer from abdominal pain after taking colchicine. Furthermore, she did not suffer from abnormal uterine bleeding, and anemia was resolved by taking sodium ferrous citrate. Nevertheless, the recovery from acute renal failure was not sufficient even after relieving the inflammatory response and we consulted with a nephrologist at a nearby university hospital.

## Discussion

FMF is an autosomal-recessive inherited autoinflammatory disease, which is characterized by recurrent bouts of fever and serositis. FMF is caused by mutations in *MEFV*, which codes for the inflammation control protein "pyrin". Many cases have been reported of *MEFV *gene-related disease (e.g., arthritis, enterocolitis, meningitis, pulmonary disease, and pericarditis) [6-10]. These cases presented with recurrent serositis similar to FMF without periodic fever.

Serositis related to FMF may result in abdominal, chest, and joint pain due to peritonitis, pleuritis, pericarditis, and synovitis, respectively. It is well known that recurrent bouts of symptoms in female patients with FMF are related to menstruation [11-13]. Although male patients with FMF may present with acute scrotum, diagnostic criteria of FMF do not include adnexal serositis or the inflammation of uterine appendages.

Kishida et al. reported that menstruation was the most common triggering factor in female patients with FMF [14]. Furthermore, they suggested that patients with menstruation-related FMF had a younger age of onset and diagnosis, a higher frequency of peritonitis, and a higher rate of endometriosis compared with patients with non-menstruation-related FMF. It is also well known that female patients with FMF have infertility complications [15]. Okuyucu et al. examined the gravida and the changes in the ovarian reserve of Turkish patients with FMF [16]. They reported that, among the 40 patients with FMF, 16 patients had pelvic fluid, and five patients had hydrosalpinx detected by transabdominal pelvic sonography. Therefore, sonographic imaging may help diagnose female patients with FMF.

FMF and associated amyloidosis may cause both male and female infertility, but it is controversial whether colchicine treatment can relieve infertility. Hara et al. discussed the treatment strategies for patients with menstruation-induced FMF [17]. Patients with FMF have subclinical inflammations even during remissions. For preventing subclinical inflammation, a daily continuous administration of colchicine is recommended even during remissions. On the other hand, colchicine sometimes causes side effects (eg, convulsions, abdominal pain, hyperperistalsis, diarrhea, and vomiting). Intermittent colchicine therapy or an increase in the daily dose of colchicine during menstruation is the alternative therapy for patients with intolerance of colchicine.

## Conclusions

In this report, we described a patient with a *MEFV* gene variant, who experienced transient hydrosalpinx caused by adnexal serositis. Patients who experienced dysmenorrhea may have a variant gene of *MEFV* and be responsive to colchicine. Internal medicine physicians need to cooperate with gynecologists to diagnose female patients carrying *MEFV* gene variants.
